# Epidemiology, health-related quality of life and economic burden of binge eating disorder: a systematic literature review

**DOI:** 10.1007/s40519-014-0173-9

**Published:** 2015-01-09

**Authors:** Tamás Ágh, Gábor Kovács, Manjiri Pawaskar, Dylan Supina, András Inotai, Zoltán Vokó

**Affiliations:** 1 Syreon Research Institute, Thököly Street 119, 1146 Budapest, Hungary; 2 Shire Development LLC, 735 Chesterbrook Boulevard, Wayne, PA 19087 USA; 3 Department of Health Policy and Health Economics, Eötvös Loránd University, PázmányPéter Street 1/a, 1117 Budapest, Hungary

**Keywords:** Binge eating disorder, Systematic review, Epidemiology, Quality of life, Burden of illness

## Abstract

**Purpose:**

To perform a systematic review on the epidemiology, the health-related quality of life (HRQoL) and economic burden of binge eating disorder (BED).

**Methods:**

A systematic literature search of English-language articles was conducted using Medline, Embase, PsycINFO, PsycARTICLES, Academic Search Complete, CINAHL Plus, Business Source Premier and Cochrane Library. Literature search on epidemiology was limited to studies published between 2009 and 2013. Cost data were inflated and converted to 2012 US$ purchasing power parities. All of the included studies were assessed for quality.

**Results:**

Forty-nine articles were included. Data on epidemiology were reported in 31, HRQoL burden in 16, and economic burden in 7 studies. Diagnosis of BED was made using 4th Edition of The Diagnostic and Statistical Manual of Mental Disorders (DSM-IV) criteria in 46 studies. Lifetime prevalence of BED was 1.1–1.9 % in the general population (DSM-IV). BED was associated with significant impairment in aspects of HRQoL relating to both physical and mental health; the Short Form 36 Physical and Mental Component Summary mean scores varied between 31.1 to 47.3 and 32.0 to 49.8, respectively. Compared to individuals without eating disorder, BED was related to increased healthcare utilization and costs. Annual direct healthcare costs per BED patient ranged between $2,372 and $3,731.

**Conclusions:**

BED is a serious eating disorder that impairs HRQoL and is related to increased healthcare utilization and healthcare costs. The limited literature warrants further research, especially to better understand the long-term HRQoL and economic burden of BED.

**Electronic supplementary material:**

The online version of this article (doi:10.1007/s40519-014-0173-9) contains supplementary material, which is available to authorized users.

## Introduction

Binge eating disorder (BED) is a psychiatric disorder, characterized by recurrent binge eating episodes which are not followed by inappropriate compensatory behaviors. BED appears to affect a broader spectrum of the population than anorexia nervosa (AN) and bulimia nervosa (BN) [1–4], resulting in a clinically significant disorder [5].

BED was originally introduced in the 4th Edition of The Diagnostic and Statistical Manual of Mental Disorders (DSM-IV) as a sub-category of eating disorders not otherwise specified (EDNOS) [6]. In the new DSM-5, published in May 2013, BED was included as a full diagnostic entity [7]. In the DSM-IV, for a provisional diagnosis of BED, binge eating must occur on at least 2 days per week over a 6-month period [6]. In contrast, according to the new DSM-5 criteria, BED is characterized by at least one binge eating episode per week for three months [7].

The aim of this study is to undertake a systematic review of the published literature on the epidemiology, HRQoL and economic burden of BED. To our knowledge, no comprehensive review encompassing these topics has been published to date. However, this information consolidated in a single literature review provides a holistic overview of the public health importance of BED.

## Materials and methods

This review of evidence on the burden of BED was a part of a comprehensive literature review on the epidemiology, and the HRQoL and economic burden of eating disorders. A systematic literature search was conductedin July 2013 using Medline and Embase (via Scopus), PsycINFO, PsycARTICLES, Academic Search Complete, CINAHL Plus, Business Source Premier (via Ebsco Host) and Cochrane Library. Search terms were combinations of terms related to the disease (BED, AN, BN and EDNOS), epidemiology (prevalence, incidence and mortality), HRQoL burden (quality of life, health burden, humanistic burden, quality adjusted life years and disability adjusted life years) and economic burden (direct healthcare costs, direct patient and caregiver cost, wider societal cost). For case of studies related to HRQoL and/or economic burden, there were no pre-defined publication date limits. To get up to date epidemiological data, literature search on epidemiology was limited to studies published between 2009 and 2013. Specification of the review protocol is provided in Online Resource 1.

Search results were considered in two steps. Initially, titles and abstracts of all articles were screened using the following inclusion criteria: (1) the article was written in English; (2) the article was related to eating disorders; and (3) the article studied the epidemiology, HRQoL burden and/or economic burden of eating disorders. Those articles deemed relevant were analyzed in full text. During the full text review only publications in which BED was defined using either the DSM-IV or DSM-5 criteria (proposed or published), and BED sample was clearly separated from other eating disorders were included; reasons for exclusion were: (1) the article was not written in English; (2) the article was not published in peer-reviewed journals; (3) the article was editorial, letter, case report or review; (4) the article was not specific to BED; or (5) the article had other objective than studying the epidemiology, HRQoL and/or economic burden of BED. References of relevant articles were screened for additional eligible studies. Literature screening was conducted by two reviewers independently; disagreements were resolved by the principal researcher.

Data extraction focused on and was limited to findings relevant to the research topic. The following information was extracted from each study: (1) first author and year of publication; (2) country; (3) study design; (4) study year; (5) included eating disorder(s); (6) diagnostic method and criteria for BED; (7) characteristics of study sample [sample size,  % of female, mean age, mean body mass index (BMI)]; (8) epidemiological data [incidence, prevalence (lifetime, 12-month, point), mortality, suicide (ideation, attempt)]; (9) data on the HRQoL burden of BED (HRQoL instrument, HRQoL data) (10) data on healthcare utilization of BED patients (data source, resource utilization categories, main findings); and (11) data on healthcare costs and/or societal costs related to BED (perspective of the analysis, data source, year of pricing, cost categories, cost data). For interventional studies, in which HRQoL was assessed, only the baseline HRQoL data were extracted as this review investigated the association between BED and patient’s HRQoL and not the efficacy/effectiveness of different interventions on HRQoL in BED.

In this review, prevalence and incidence were defined as follows: incidence as the number of new cases of a disorder in the population over a specified period related to the number of persons at risk at the beginning of the period (cumulative incidence) or to the person-time of observation (incidence rate); point prevalence as the proportion of persons affected with the disorder at a specific point in time; 12-month prevalence rate as the point prevalence plus the annual cumulative incidence (the proportion of people with new cases in the following year); lifetime prevalence as the proportion of people that had the disorder at any point in their life.

In order to compare cost data across studies, costs were extrapolated to annual costs per patient and were inflated to the year of 2012 using country specific gross domestic product (GDP) deflators, and converted into United States (US) dollars ($), using purchasing power parities. If the year of pricing was not referenced, then the midpoint in the observation period was assumed as the base year. If no observation period was reported, the year of publication was adopted.

Quality assessment of the included studies was performed using the strengthening the reporting of observational studies in epidemiology (STROBE) checklist for cohort, case–control, and cross-sectional studies [8]. The results for each study were summarized as the percentage of the fulfilled criteria. STROBE criteria that were not applicable to a study were excluded from the quality assessment. All studies were independently assessed by two researchers, disagreements were resolved by the principal researcher.

## Results

### Search results

**Fig. 1:**
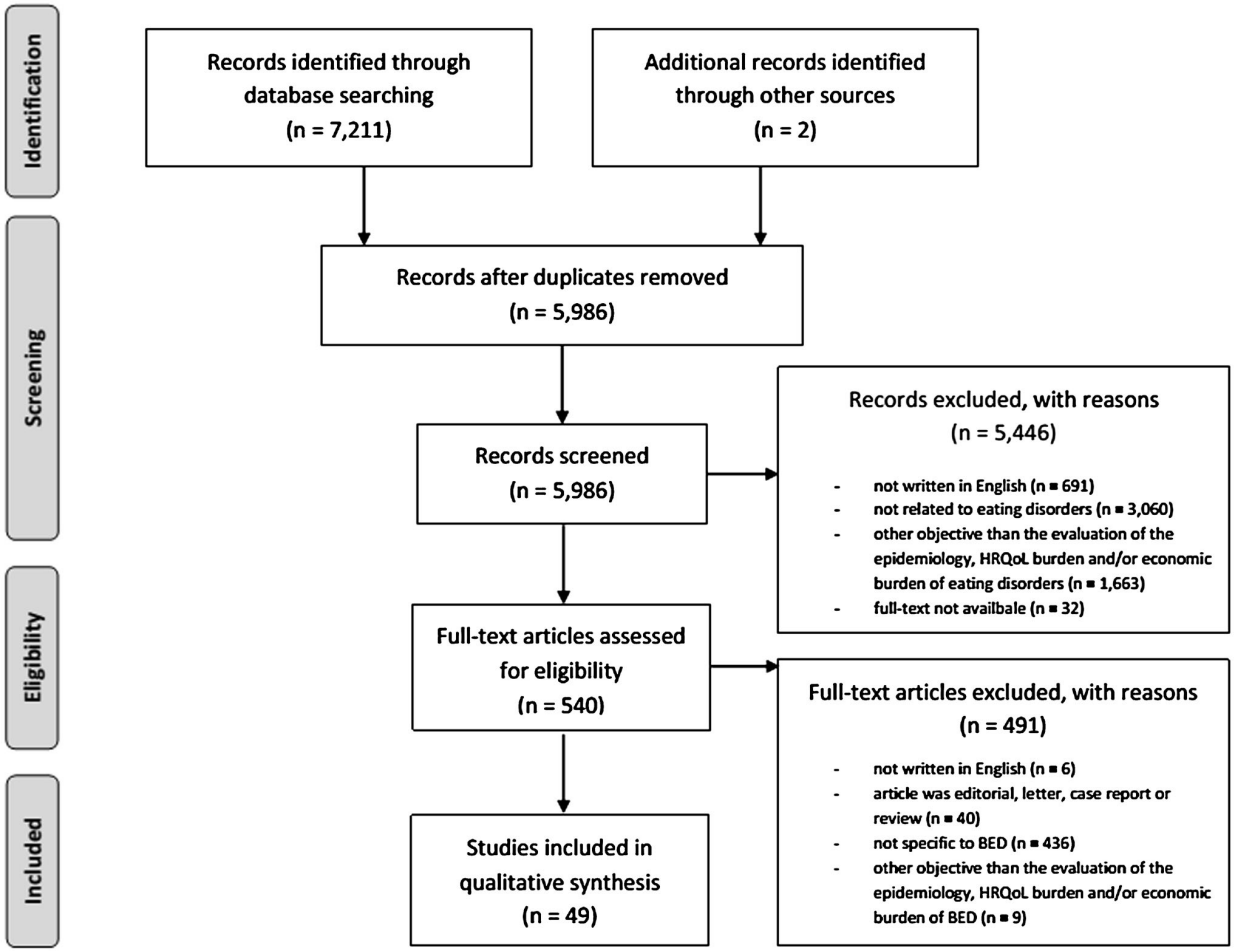
The flow diagram of the systematic literature search. *BED* binge eating disorder. *HRQoL* health-related quality of life

The database search resulted in 7,211 hits. Screening of titles and abstracts identified 540 potentially eligible articles. Two additional records were identified through hand search of the references of relevant articles. After the review of relevant full text articles, 49 studies were included in this systematic review. The flow diagram of the systematic literature search, based on the preferred reporting items for systematic reviews and meta-analyses (PRISMA) template [9], is presented in Fig. 1.

Most studies (*n* = 40) were cross-sectional [2–4, 19–55], 7 longitudinal [10–12, 15–18], and 2 were database analyses [13, 14]. Included studies originated from 23 countries, with over half (*n* = 25) conducted in the US. The sample sizes of the included studies ranged from 31 [19] to 77,807 [12], with a mean ages from 13.0 [16] to 49.8 [20] years. Many studies only included females (*n* = 18) but in those including both sexes, females were over-represented. Diagnosis of BED was made using DSM-IV criteria in 44 [2–4, 10, 12–15, 17–20, 22–30, 32–41, 43–55], using the proposed DSM-5 criteria in 3 [11, 16, 31], and using both DSM-IV and the proposed DSM-5 criteria in 2 studies [21, 42]. The general characteristics of the reviewed studies are presented in Online Resource 2.

Included studies (*n* = 49) fulfilled between 26.5 [21] and 84.4 % [3] of the STROBE criteria. Details of the quality assessment are provided in Online Resource 3.

### Epidemiology

Only one study reported incidence rate for BED [16]. A sample of 496 adolescent females over an 8-year period found the incidence of BED (DSM-5) to be 343 per 100,000 person-years [16].

Point prevalence rate for BED was reported in 22 studies [12, 14, 20–39], 12-month prevalence rate in 4 studies [2–4, 13] and lifetime prevalence rate in 11 studies [2–4, 13, 16, 17, 21, 36, 40–42]. Detailed information on the reported prevalence rates are presented in Online Resource 2. Point prevalence rates, not limited to population-based estimates varied between 0.1 % (in a sample of pregnant women, 6–12 months prior pregnancy) [27] and 34.1 % (in a sample with type 2 diabetes) [20] using DSM-IV criteria, and 0.6 % (in a sample of female high school and university students) [31] to 1.7 % (in a sample of first degree relatives of BED patients) [21] using the proposed DSM-5 criteria. The 12-month prevalence rate of BED, not limited to population-based estimates ranged between 0.1 % (in a large community sample of people over 18 years of age) [3] and 1.1 % (in a sample of Latino) [13] using DSM-IV criteria. Lifetime prevalence for BED, not limited to population-based estimates was 0.17 % (in a sample of adult female twins) [42] to 8.8 % (in a sample of outpatients with bipolar disorder) [40] using DSM-IV criteria and 0.2 % (in a sample of adult female twins) [42] to 3.6 % (in a sample of first degree relatives of BED patients) [21] using the proposed DSM-5 criteria.

In three studies the lifetime prevalence of BED (DSM-IV) was 1.5–6 times higher in women than in men [3, 4, 21]. Hudson et al. [21]. found no difference in the proportion of women and men regarding the DSM-IV and the proposed DMS-5 lifetime prevalence of BED (Online Resource 2). Sex difference was also observed in point and 12-month prevalence rates (Online Resource 2). The majority of BED cases occurred first in people’s lives between the ages of 12.4 and 24.7 years [3, 4, 16, 43]; however, the prevalence of BED continued to rise until 40 years old [3]. From the reviewed articles information on ethnic or racial differences in prevalence of BED was limited and inconsistent. Azarbard et al. [24] found no significant differences in the prevalence of BED among Hispanic, African American and White women (Online Resource 2). However, Perez et al. [14] reported higher prevalence for BED among Hispanic (2.3 %) and Black individuals (1.6 %) compared to White individuals (1.0 %).

Several studies examined the association of BED with physical and psychiatric comorbidities. In the study Kessler et al. [2] the odds ratio (OR) for BED was 2.9 for individuals with diabetes, 2.2 for hypertension, 1.6 for stroke and 1.3 for heart disease (population-based estimates). Point prevalence of BED (DSM-IV) in overweight/obese individuals was reported to be 5.9 % in a sample with serious mental illness [30], 13.4 % in a sample waiting for bariatric surgery [39] and 23.9 % in a sample seeking weight loss treatment [34]. The OR for lifetime BED was 0.7 for BMI < 18.5, 1.0 for BMI 18.5–24.9, 1.3 for BMI 25–29.9, 3.1 for BMI 30–34.9, 3.0 for BMI 35–39.9 and 6.6 for BMI > 40 (population-based estimates) [2]. Lifetime prevalence of BED was found to be elevated in patients with obsessive–compulsive disorder and bipolar I or II disorders [36, 40]. Compared to a non-eating disorder group, the mean scores in Beck anxiety inventory was higher for the BED group (23 vs. 13, *p* < 0.001) and for the Beck depression inventory (23 vs. 15, *p* < 0.001) [36]. Several studies reported that BED occurred in a significant number of women during pregnancy (1.8–7 %) and in the post-partum period (2.7–3.1 %) [12, 27, 28, 32].

Mortality for BED was not reported in any of the included studies and only 3 studies described data on suicide attempt and/or ideation in BED patients [4, 22, 44]. When comparing asymptomatic and BED individuals, among a school-based sample of youth the ORs for suicidal ideation and attempt were 2.6 and 3.1, respectively [22]. In the studies conducted by Carano et al. [44] and Swanson et al. [4], 27.5 % (in adults) to 34.4 % (in adolescents) of individuals with BED had suicidal ideations and the frequency of suicidal attempts was 12.5 % (in adults) to 15.1 % (in adolescents).

### Health-related quality of life burden

Sixteen studies reported data on the HRQoL burden of BED (Table 1) [10, 11, 14, 15, 18, 19, 34, 43, 45–52].Various questionnaires were used to measure HRQoL in the studies. All but one study used validated HRQoL questionnaires (Table 1). Perez et al. [14] used a self-developed instrument. General HRQoL measures included the Extended Satisfaction With Life Scale (ESWLS) [10], EuroQol Five Dimensional Questionnaire (EQ-5D-3L) [43], Short Form-36 (SF-36) [11, 19, 45–48, 50], Short Form-12 (SF-12) [49] or World Health Organization Brief Quality of Life Assessment (WHOQOL-BREF) [15, 49]. The SF-36 (*n* = 7) was the most commonly used HRQoL questionnaire. An obesity-specific measure was used in 5 studies (Impact of Weight on Quality of Life Lite (IWQOL-LITE) [18, 45, 51, 52] and Obesity Related Well-Being (ORWELL) [34]).

**Table 1:** Summary of the health related quality of life data of the included studies

First author, reference	HRQoL instrument	Study sample	Sample size	HRQoL data, baseline mean (SD)
Cassin [10]^a^	ESWLS	BED, AMI	54	ESWLS General life score: 16.5 (7.8), ESWLS Social life score: 14.1 (8.0), ESWLS Sex life score: 12.0 (8.0), ESWLS Self score: 14.1 (6.5), ESWLS Physical appearance score: 7.1 (3.9), ESWLS Family score: 19.5 (8.8), ESWLS Relationships score: 17.5 (10.1)
		BED, control (no AMI)	54	ESWLS General life score: 17.1 (8.0), ESWLS Social life score: 15.8 (8.5), ESWLS Sex life score: 13.5 (8.7), ESWLS Self score: 16.0 (6.9), ESWLS Physical appearance score: 8.7 (4.9), ESWLS Family score: 18.4 (10.1), ESWLS Relationships score: 19.2 (9.3)
De Zwaan [45]^b^	SF-36	BED, postoperative	9	SF-36 PCS: 42.2 (8.9), SF-36 MCS: 45.7 (10.4)
		No BED, postoperative	69	SF-36 PCS: 47.2 (10.2), SF-36 MCS: 52.8 (7.8)
De Zwaan [46]^b^	SF-36	BED, preoperative	19	SF-36 PCS: 31.1 (9.2), SF-36 MCS: 49.8 (10.1)
		No BED, preoperative	91	SF-36 PCS: 28.1 (6.8), SF-36 MCS: 44.7 (9.5)
	IWQOL-LITE	BED, preoperative	19	IWQOL-LITE total score: 46.7 (17.6)
		No BED, preoperative	91	IWQOL-LITE total score: 34.0 (9.9)
Doll [47]^b^	SF-36	BED	18	SF-36 PCS: 46.3 (9.1), SF-36 MCS: 40.8 (9.0)
		AN	6	SF-36 PCS: 43.4 (9.0), SF-36 MCS: 45.54 (8.9)
		BN	45	SF-36 PCS: 47.2 (9.5), SF-36 MCS: 43.8 (9.4)
		No ED	1,219	SF-36 PCS: 48.1 (16.7), SF-36 MCS: 49.8 (16.5)
Faulconbridge [11]^a^	SF-36	BED, bariatric surgery	36	SF-36 PCS: 37.7 (1.7), SF-36 MCS: 43.1 (1.6)
		BED, lifestyle modification	49	SF-36 PCS: 40.8 (1.3), SF-36 MCS: 45.4 (2.0)
Grenon [43]^b^	EQ-5D-3L	BED	105	EA-5D-3L index score: 0.77 (0.2)
Hsu [19]^b^	SF-36	BED	37	SF-36 PCS: 33.0^d^, SF-36 MCS: 45.0^d^
Kolotkin [52]^b^	IWQOL-LITE	BED	95	IWQOL-LITE total score: 51.5 (21.9)
		No BED	435	IWQOL-LITE total score: 65.3 (19.8)
Masheb [48]^b^	SF-36	BED	94	SF-36 PCS: 47.3 (10.2), SF-36 MCS: 39.7 (11.0)
		BED, BMI ≥ 30	71	SF-36 PCS: 45.3 (9.6), SF-36 MCS: 39.3 (10.6)
		BED, BMI < 30	23	SF-36 PCS: 53.6 (9.4), SF-36 MCS: 41.0 (12.2)
Mond [49]^b^	SF-12	BED	10	SF-12 PCS: 40.2 (13.1), SF-12 MCS: 30.4 (8.0)
		AN, restricting type	19	SF-12 PCS: 45.4 (10.3), SF-12 MCS: 38.4 (11.1)
		AN, purging type	15	SF-12 PCS: 46.8 (10.0), SF-12 MCS: 27.0 (7.4)
		BN	40	SF-12 PCS: 49.3 (10.1), SF-12 MCS: 27.6 (9.4)
		Normal control (no ED)	495	SF-12 PCS: 50.7 (8.8), SF-12 MCS: 47.4 (10.3)
	WHOQOL-BREF	BED	10	WHOQOL-BREF QoLP: 2.2 (0.5), WHOQOL-BREF QoLS: 2.2 (1.0)
		AN, restricting type	19	WHOQOL-BREF QoLP: 2.7 (0.9), WHOQOL-BREF QoLS: 3.6 (1.0)
		AN, purging type	15	WHOQOL-BREF QoLP: 2.1 (0.7), WHOQOL-BREF QoLS: 2.6 (1.1)
		BN	40	WHOQOL-BREF QoLP: 2.4 (0.7), WHOQOL-BREF QoLS: 3.1 (1.0)
		Normal control (no ED)	495	WHOQOL-BREF QoLP: 3.7 (0.6), WHOQOL-BREF QoLS: 3.7 (0.8)
Padierna [50]^b^	SF-36	BED	17	SF-36 PCS: 36.5^d^, MCS: 32.0^d^
		AN, restricting type	56	SF-36 PCS: 44.0^d^, SF-36 MCS: 34.0^d^
		AN, purging type	60	SF-36 PCS: 43.5^d^, SF-36 MCS: 28.0^d^
		BN	64	SF-36 PCS: 43.0^d^, SF-36 MCS: 30.0^d^
Perez [14]^c^	self-developed	Non-obese without BED	12,063	Physical health score: 2.6 (0.7), Mental health score: 2.2 (1.0)
		Non-obese with BED	124	Physical health score: 2.5 (0.8), Mental health score: 2.5 (1.2)
		Obese without BED	4,585	Physical health score: 2.6 (0.8), Mental health score: 2.2 (1.0)
		Obese with BED	126	Physical health score: 2.4 (0.8), Mental health score: 2.8 (1.1)
Ricca [34]^b^	ORWELL	BED	105	ORWELL total score: 54.3 (21.2)
		BED subthreshold	146	ORWELL total score: 53.0 (20.3)
		Overweight non-BED	187	ORWELL total score: 56.0 (22.2)
Rieger [51]^b^	IWQOL-LITE	BED	56	IWQOL-LITE total score: 74.0 (19.3)
		No BED	62	IWQOL-LITE total score: 61.2 (26.3)
Silveria [15]^a^	WHQOL-BREF	BED	9	n.r.
Wilfley [18]^a^	IWQOL-LITE	BED, sibutramine	152	IWQOL-LITE total score: 67.7 (18.2)
		BED, placebo	152	IWQOL-LITE total score: 68.7 (18.5)

*AMI* adapted motivational interviewing, *AN* anorexia nervosa, *BED* binge eating disorder, *BN* bulimia nervosa, *ESWLS* Extended Satisfaction With Life Scale, *EQ-5D-3L* EuroQol Five Dimensional Questionnaire, *HRQoL* health-related quality of life, *IWQOL-LITE* Impact of Weight on Quality of Life–Lite, *MCS* mental component summary, *n.r*. not reported, *ORWELL* obesity related well-being, *PCS* physical component summary, *QoLP* psychological health, *QoLS* social relationships, *SD* standard deviation, *SF-12* Short-Form Disability Scale, *SF-36* Short Form 36, *WHOQOL-BREF* World Health Organization Brief Quality of Life Assessment Scale

^a^Longitudinal study

^b^Cross-sectional study

^c^Retrospective data analysis

^d^HRQoL data were presented only in figure form, HRQoL data were extracted from the figure

HRQoL of patients with BED was significantly lower than in control subjects (Table 1) [14, 34, 45–47, 49, 51, 52]. BED was associated with marked impairment compared to general population norms in both the physical component summary (PCS) and the mental component summary (MCS) scores of SF-36 (Masheb et al. [48] PCS_BED_: 47.3, MCS_BED_: 39.7; Padierna et al. [50]. PCS_BED_: 36.5, MCS_BED_: 32.0 vs. PCS_population norm_ = 50.0, MCS_population norm_ = 50.0). In the study conducted by Grenon et al. [43] the EQ-5D-3L mean score of overweight/obese women with BED (0.77) was significantly lower than for a US community sample of women with a similar mean age (0.89).

Where studies evaluated HRQoL for the different eating disorders, there were no significant differences among AN, BN and BED (Table 1) [47, 49, 50]; but, decreased physical HRQoL appeared to be most evident in patients with BED. In the study by Mond et al. [49] physical health measured by SF-12 was poorer in BED (PCS: 40.2) than in AN (PCS_restricting type_: 45.4, PCS_purging type_: 46.8) and BN (PCS: 49.3); however, most BED patients were obese (BMI ≥ 30) and had a higher mean age in that patient group [mean age (standard deviation); AN restricting type: 19.31 (4.22); AN purging type: 25.53 (9.77); BN: 23.48 (6.25); BED: 34.33 (7.37)]. These findings were consistent with those of Padierna et al. [50], who reported more impaired physical health (SF-36) in BED patients (PCS: 36.5); nevertheless the differences compared to AN (PCS_restricting type_: 44.0, PCS_purging type_: 43.5) and BN (PCS: 43.0) were not statistical significant (Padierna et al. [50] presented their results only in figure form, SF-36 scores were extracted from the figure).

Among subgroups of BED, obese BED patients had significantly worse HRQoL than non-obese BED patients (Table 1) [14, 34, 49]. In addition, BED was associated with more impairment in HRQoL than obesity without BED (IWQOL-LITE BED: 74.0 vs. No BED: 61.2; higher scores indicating greater impairment) [51]. Obesity status more strongly affected the physical dimension of HRQoL, whereas BED status affected mental health and social functioning HRQoL [14]. Among BED patients, obese BED subjects (PCS: 45.3) had significantly lower scores in PCS scores on the SF-36 compared to non-obese (PCS: 53.6) [48]. Depressive symptoms were also reported to be significantly associated with lower HRQoL in patients with BED (measured with EQ-5D-3L, after controlling for age and BMI) [43].

### Economic burden

Healthcare utilization in BED was assessed only in 8 studies [2–4, 13, 43, 53–55]. Healthcare costs were reported only in two studies [43, 53]. Reported healthcare utilization and healthcare costs data are presented in Table 2 and Table 3.

**Table 2:** Selected healthcare utilization data for patients diagnosed with binge eating disorder

First author, reference	Sample size	Reported healthcare utilization data	
Dickerson [53]	BED sample (*n*): 50	*% with any use*	
		Weight- and eating disorder-related services	24
		Non-weight- and eating disorder-related mental health services	24
		Other provider-based services	100
		Mental health medication services	62
		Total medication services	90
		Total health services	100
Grenon [43]	BED sample (*n*): 105	*% of participants endorsing each health care domain*	
		Family physician visits	82
		Medication use	72
		Diagnostic tests	53
		Health professionals’ visits	51
		Specialist visits	49
		Herbal remedies	34
		Other resources	16
		Out-patient visits	15
		Emergency department visits	13
		In-patient visits	2
Kessler [2]	Total sample (*n*): 24,124 BED, lifetime prevalence: 1.9 %	Lifetime treatment for emotional problems (%)	58
		12-month treatment for emotional problems (%)	37
		Lifetime treatment for eating disorders (%)	38
		12-month treatment for eating disorders (%)	10
Marques [13]	Total sample (*n*): n.r. BED, lifetime prevalence:	*Lifetime any service use* (%)	
	Latino: 2.1 %	Non-Latino White	79
	Non-Latino White: 1.4 %	Latino	54
	African American: 1.5 %	Asian	55
	Asian: 1.2 %	African American	71
Mond [54]	BED sample (*n*): 20	*Lifetime service use* (%)	
		Any treatment	
		Eating	58
		General mental health	84
		Weight	87
		Treatment by a mental health professional	
		Eating	23
		General mental health	39
		Weight	45
Preti [3]	Total sample (*n*): 21,425 BED, lifetime prevalence: 1.1 %	*Lifetime access to service use for any emotional problem* (%)	
		General medical	24
		Psychiatrist	16
		Other mental health	18
		Non-medical professional	6
		Complementary-alternative medical	12
		Any lifetime treatment	39
		*12*-*month access to service use for any emotional problem* (%)	
		General medical	17
		Psychiatrist	10
		Other mental health	3
		Non-medical professional	10
		Complementary-alternative medical	1
		Any 12-month treatment	23

First author, reference	Sample size	Reported healthcare utilization data												
Striegel-Moore [55]	BED sample (*n*): 162	**12-month data**												
			Binge eating disorder				Healthy comparison				Psychiatric comparison			
			White		Black		White		Black		White		Black	
			Obese	Non-obese	Obese	Non-obese	Obese	Non-obese	Obese	Non-obese	Obese	Non-obese	Obese	Non-obese
		Out-patient psychotherapist visits %	33	24	24	27	0	3	0	7	54	56	25	23
		Emergency department visits %	40	24	16	33	17	13	14	14	23	21	38	62
		In-patient days %	11	11	7	13	11	8	14	4	31	10	25	23
		Out-patient physician visits mean (SD)	7.9 (10.5)	4.1 (5.8)	4.4 (8.2)	7.1 (10.1)	6.2 (8.6)	3.1 (3.6)	4.1 (7.7)	3.7 (5.3)	8.2 (10.8)	6.0 (8.1)	3.3 (3.0)	4.4 (4.4)
		Total service days mean (SD)	21.4 (28.1)	11.8 (21.8)	17.6 (30.1)	14.9 (23.0)	8.4 (12.7)	3.4 (3.9)	4.6 (8.4)	4.8 (7.9)	16.1 (14.0)	18.4 (23.7)	6.9 (4.6)	8.5 (10.8)
Swanson [4]	Total sample (*n*): 10,123 BED lifetime prevalence: 1.6 %	**Lifetime service use**												
							Binge eating disorder (%)				No-eating disorder (%)			
		Mental health specialty					60				29			
		General medicine					22				12			
		Human service					18				10			
		Complementary and alternative medicine					4				8			
		Juvenile justice					1				5			
		School service					20				21			
		Any treatment					73				44			
		Treatment specifically for eating or weight problems					11				n.a.			

*n.r.* not reported, *n.a*. not applicable, *SD* standard deviation

**Table 3:** Selected direct healthcare cost data for patients diagnosed with binge eating disorder

Reference	Country, year of pricing	Cost categories and reported costs			Annual costs per patient in 2012 $	
Dickerson [53]	US 2006	**Binge eating disorder**				
		Total costs mean (SD)	$3,319 (4,050)		$3,731	
		Weight- and eating disorder-related services mean (SD)	$72 (176)			
		Non-weight- and eating disorder-related mental health services mean (SD)	$415 (1,014)			
		Other provider-based services mean (SD)	$1,925 (2,761)			
		Mental health medication services mean (SD)	$411 (695)			
		Total medication services mean (SD)	$906 (1,475)			
		**Recurrent binge eating** ^a^				
		Total costs mean (SD)	$3,588 (4,665)		$4,033	
		Weight- and eating disorder-related services mean (SD)	$94 (299)			
		Non-weight- and eating disorder-related mental health services mean (SD)	$341 (840)			
		Other provider-based services mean (SD)	$2,221 (3,818)			
		Mental health medication services mean (SD)	$301 (637)			
		Total medication services mean (SD)	$933 (2,087)			
Grenon [43]	Canada 2009	Total cost^b^ (6-month) mean (SD)	Can$1,379 (1,252)		$2,372	

*Can$* Canadian dollar, *$* United States dollar, *SD* standard deviation, *US* United States

^a^Individuals with at least 1 day per week with an objective bulimic episode over a 3-month period, with no periods of binge free time greater than 2 weeks, and did not meet DSM-IV criteria for bulimia nervosa or binge eating disorder

^b^Family physician visits, medication use, diagnostic tests, health professionals’ visits, specialist visits, herbal remedies, other resources, out-patient visits, emergency department visits, and in-patient visits

Striegel-Moore et al. [55] analyzed the healthcare utilization in women with BED using emergency room visits, outpatient physician visits for medical care, outpatient psychotherapy visits and days spent in the hospital over the last 12 months. Compared to healthy individuals (mean total service days: 3.4–8.4), BED was associated with increased total health service use (mean total service days: 11.8–21.4), but resource utilization in BED was similar to other major psychiatric conditions (mean total service days: 6.9–18.4) (Table 2). There was no significant difference in resource utilization between BED group and non-eating Axis I psychiatric disorders group.

In the included studies, only a minority of BED patients received treatment specifically for their eating disorder. Swanson et al. [4] found that the lifetime service use for treatment for eating or weight problems among BED patients was only 11.9 %. In the study conducted by Kessler et al. [2] 38.3 % of patients with a lifetime diagnosis of BED had received treatment specifically for an eating disorder. Mond et al. [54] reported that in their study sample, 58.1 % of BED patients had received treatment for an eating problem, but 83.9 % received treatment for general mental health and 87.1 % for weight loss. Only 12.9 % of BED patients were treated by a mental health professional specifically for their eating disorder.

Marques et al. [13] examined mental health service utilization for differences among ethnic groups. They found mental health service use was higher for non-Latino Whites (78.9 %) than for Latinos (54.1 %), Asians (54.5 %) or African Americans (71.1 %).

The reported annual direct healthcare costs per BED patient ranged from a low of $2,372 [43] to a high of $3,731 [53] (Table 3). In one Canadian study [43], the annual healthcare cost of overweight/obese women with BED was 36.5 % higher than the estimated health expenditure per capita for women in the same age group. Another US study [53] compared healthcare utilization between women with BED (DSM-IV) and recurrent binge eating (individuals with at least 1 day per week with an objective bulimic episode over a 3-month period, with no periods of binge free time greater than 2 weeks, and did not meet DSM-IV criteria for BN or BED), and evaluated the effect of the number of binge eating days on healthcare costs. BED and recurrent binge eating groups did not differ significantly in total annual healthcare costs adjusted for age, BMI and self-reported depression.

## Discussion

This systematic review of the epidemiology, and the HRQoL and economic burden of BED consolidated existing data and provides a holistic overview of impact of BED on public health. It also exposes the gaps in available data where future studies might enable a better understanding of the societal costs and overall burden of BED.

We analyzed 49 studies assessing the epidemiology, economic and HRQoL burden of BED. The methodological quality of the included studies was heterogeneous. Issues related to statistical analysis such as sensitivity analysis and addressing missing data were frequently neglected.

Based on mixed-gender surveys, the lifetime prevalence of BED appears to be about 1–2 %, with the 12-month prevalence rate of 0.1–1 % in the general population (DSM-IV) [2–4]. Point prevalence for BED was assessed in many studies; however, the results of these prevalence studies are difficult to compare because studies targeted different study populations, and used different instruments for screening for BED. Hudson et al. [1] reported that using the proposed DSM-5 criteria instead DSM-IV criteria might increase the lifetime prevalence of BED with 2.9 % in women and 3.0 % in men. In consistent with these findings, Trace et al. [42] also assessed higher lifetime prevalence using the proposed DSM-5 criteria (DSM-IV: 0.17 % vs. proposed DSM-5: 0.20 %). BED appears to be 1.5–6 times more prevalent among females than males [3, 4, 21].BED can affect people of any age; but people in their 20s have higher risk than cohorts of other age groups [3].

BED is found to co-occur with several mental (anxiety, depression, obsessive–compulsive disorder) and somatic disorders (diabetes, hypertension) [2, 36, 40]. Pregnancy and the post-partum period may be periods of particularly high-risk for BED [12, 27, 28, 32]. Findings suggest that pregnancy may not only facilitate the recurrence of BED, but even contribute to its onset [28].

There have been no published studies investigating the effects of BED on mortality, although it is comorbid with disorders that are known to increase mortality risks. More systematic assessment of the potential contribution of BED status to mortality risks would be of value. Some research did focus on suicidal ideations and attempts of patients with BED [4, 22, 44]. This may suggest BED may be associated with elevated levels of suicidal ideations and even attempts compared to non-BED controls [22]. Suicidal ideations in patients with BED appears to be 27.5 % (in adults) to 34.4 % (in adolescents) with the frequency of suicidal attempts 12.5 % (in adults) to 15.1 % (in adolescents) [4, 44].

Comorbid BED is a common problem in the obese population [2]. Obesity, especially morbid obesity (BMI > 40) accounts for significant impairment in HRQoL in BED [14, 34, 48]. Obesity is associated with diabetes, hypertension, hypercholesterolemia, heart failure, ischemic heart disease and may have severe health consequences over time. Studies to increase understanding the long-term burden of BED in these patients are needed. Currently we lack data on the long-term health impact of BED. Obesity is also related to the increased risk for mortality, and though it may be difficult to quantify the net effect of BED on obesity and on the consequential mortality, these are important research questions.

BED significantly affects HRQoL. In BED, both physical and mental dimensions of HRQoL were significantly below population norms, with the mental component affected more [48, 50]. Compared to other eating disorders, BED seemed to have lower (statistically not significant) ratings in physical HRQoL [49, 50], which may be partially accounted for by overweight status and poor physical health [49].

Although BED is a prevalent eating disorder, there is very limited literature examining the economic burden (*n* = 8) of BED [2–4, 13, 43, 53–55]. Additionally, available literature on its economic burden has used inconsistent measures, limiting our ability to draw reliable conclusions. BED is associated with a high rate of hospitalization, outpatient care, and emergency department use [55]. BED patients rarely utilize the appropriate healthcare services specifically for their eating disorder; patients mostly receive treatment for general mental health and/or weight loss [2, 4, 54]. This makes it difficult to sufficiently gauge the relative impact of BED on these costs. This may be partly explained in that healthcare providers have not recognized BED as disorder requiring a specific treatment. Improved diagnosis, awareness and treatment may eventually provide better data for estimating economic burden. The annual direct healthcare cost in BED appears to be higher than an age and sex matched national average [43]. Dickerson et al. [53] found no significant difference between the annual healthcare costs of patients with BED (DSM-IV) and recurrent binge eating.

The findings of this study should be considered in light of the following limitations. Our systematic review identified only studies published in English, in peer-reviewed journals that were indexed in the selected databases. In order to get the most up to date data, the epidemiological data were extracted only from studies published between 2009 and 2013 only. STROBE checklist was developed for the quality assessment of observational studies in which data were collected for the research purpose. Specific issues related to research using routinely collected data are not addressed in STROBE; nevertheless, we also used it for assessing the quality of those studies in which retrospective database analyses were performed. Included studies originated from 23 countries [with over half (*n* = 25) conducted in the US]; however, because of the low number of included articles, cultural differences could not be addressed in this review. In this review we estimated the occurrence of BED and did not study its determinants, therefore confounding was not an issue. Nevertheless, misclassification and selection bias might have occurred to some extent, since not all the studies included used the same diagnostic criteria, and the method of case ascertainment was different.

In summary, BED has a lifetime prevalence rate of 1–2 % in the general population. BED significantly affects HRQoL. It is associated with increased healthcare utilization and healthcare costs compared to individuals without an eating disorder. Obesity and especially morbid obesity account for significant impairment in BED; however, the negative health consequences of obesity can be more significant on the long run. Only a minority of patients receives a specific treatment for BED. With education of providers on the diagnosis, reporting/coding, treatment, and management of BED, data should become available to permit more accurate assessment of the HRQoL and economic burden of this disorder. Future research (i.e., longitudinal studies and randomized controlled trials) is needed to better understand long-term consequences of BED and also help with better management of this disorder.

## Electronic supplementary material

Below is the link to the electronic supplementary material.

Supplementary material 1 (PDF 277 kb)

Supplementary material 2 (PDF 397 kb)

Supplementary material 3 (PDF 308 kb)

## References

[1] Hudson JI, Hiripi E, Pope HG, Kessler RC (2007). The prevalence and correlates of eating disorders in the national comorbidity survey replication. Biol Psychiatry.

[2] Kessler RC, Berglund PA, Chiu WT, Deitz AC, Hudson JI, Shahly V, Aquilar-Gaxiola S, Alonso J, Angermeyer MC, Benjet C, Bruffaerts R, de Girolamo G, de Graaf R, Maria Haro J, Kovess-Masfety V, O’Neill S, Posada-Villa J, Sasu C, Scott K, Viana MC, Xavier M (2013). The prevalence and correlates of binge eating disorder in the World Health Organization World Mental Health Surveys. Biol Psychiatry.

[3] Preti A, Girolamo G, Vilagut G, Alonso J, Graaf R, Bruffaerts R, Demyttenaere K, Pinto-Meza A, Haro JM, Morosini P (2009). The epidemiology of eating disorders in six European countries: results of the ESEMeD-WMH project. J Psychiatr Res.

[4] Swanson SA, Crow SJ, Le Grange D, Swendsen J, Merikangas KR (2011). Prevalence and correlates of eating disorders in adolescents. Results from the national comorbidity survey replication adolescent supplement. Arch Gen Psychiatry.

[5] Wilfley DE, Wilson GT, Agras WS (2003). The clinical significance of binge eating disorder. Int J Eat Disord.

[6] American Psychiatric Association (2000). Diagnostic and statistical manual of mental disorders (4th edition, text revision).

[7] American Psychiatric Association (2013). Diagnostic and statistical manual of mental disorders.

[8] von Elm E, Altman DG, Egger M, Pocock SJ, Gotzsche PC, Vandenbroucke JP (2008). The strengthening the reporting of observational studies in epidemiology (STROBE) statement: guidelines for reporting observational studies. J Clin Epidemiol.

[9] Moher D, Liberati A, Tetzlaff J, Altman DG (2009). Preferred reporting items for systematic reviews and meta-analyses: the PRISMA statement. Ann Intern Med.

[10] Cassin SE, von Ranson KM, Heng K, Brar J, Wojtowicz AE (2008). Adapted motivational interviewing for women with binge eating disorder: a randomized controlled trial. Psychol Addict Behav.

[11] Faulconbridge LF, Wadden TA, Thomas JG, Jones-Corneille LR, Sarwer DB, Fabricatore AN (2013). Changes in depression and quality of life in obese individuals with binge eating disorder: bariatric surgery versus lifestyle modification. Surg Obes Relat Dis.

[12] Knoph C, Von Holle A, Zerwas S, Torgersen L, Tambs K, Stoltenberg C, Bulik CM, Reichborn-Kjennerud T (2013). Course and predictors of maternal eating disorders in the postpartum period. Int J Eat Disord.

[13] Marques L, Alegria M, Becker AE, Chen CN, Fang A, Chosak A, Diniz JB (2011). Comparative prevalence, correlates of impairment, and service utilization for eating disorders across US ethnic groups: implications for reducing ethnic disparities in health care access for eating disorders. Int J Eat Disord.

[14] Perez M, Warren CS (2012). The relationship between quality of life, binge-eating disorder, and obesity status in an ethnically diverse sample. Obesity (Silver Spring).

[15] Silveira RO, Zanatto V, Appolinario JC, Kapczinski F (2005). An open trial of reboxetine in obese patients with binge eating disorder. Eat Weight Disord.

[16] Stice E, Marti CN, Rohde P (2013). Prevalence, incidence, impairment, and course of the proposed DSM-5 eating disorder diagnoses in an 8-year prospective community study of young women. J Abnorm Psychol.

[17] White S, Reynolds-Malear JB, Cordero E (2011). Disordered eating and the use of unhealthy weight control methods in college students: 1995, 2002, and 2008. Eat Disord.

[18] Wilfley DE, Crow SJ, Hudson JI, Mitchell JE, Berkowitz RI, Blakesley V, Walsh BT (2008). Efficacy of sibutramine for the treatment of binge eating disorder: a randomized multicenter placebo-controlled double-blind study. Am J Psychiatry.

[19] Hsu LK, Mulliken B, McDonagh B, Krupa Das S, Rand W, Fairburn CG, Rolls B, McCrory MA, Saltzman E, Shikora S, Dwyer J, Roberts S (2002). Binge eating disorder in extreme obesity. Int J Obes Relat Metab Disord.

[20] Canan F, Gungor A, Onder E, Celbek G, Aydin Y, Alcelik A (2011). The association of binge eating disorder with glycemic control in patients with type 2 diabetes. Turk Jem.

[21] Hudson JI, Coit CE, Lalonde JK, Pope HG (2012). By how much will the proposed new DSM-5 criteria increase the prevalence of binge eating disorder?. Int J Eat Disord.

[22] Ackard DM, Fulkerson JA, Neumark-Sztainer D (2011). Psychological and behavioral risk profiles as they relate to eating disorder diagnoses and symptomatology among a school-based sample of youth. Int J Eat Disord.

[23] Anderson C, Petrie TA (2012). Prevalence of disordered eating and pathogenic weight control behaviors among NCAA Division I female collegiate gymnasts and swimmers. Res Q Exerc Sport.

[24] Azarbad L, Corsica J, Hall B, Hood M (2010). Psychosocial correlates of binge eating in Hispanic, African American, and Caucasian women presenting for bariatric surgery. Eat Behav.

[25] Bedrosian RC, Striegel-Moore RH, Wang C (2011). Demographic and clinical characteristics of individuals utilizing an internet-based digital coaching program for recovering from binge eating. Int J Eat Disord.

[26] Czarlinski JA, Aase DM, Jason LA (2012). Eating disorders, normative eating self-efficacy and body image self-efficacy: women in recovery homes. Eur Eat Disord Rev.

[27] Easter A, Bye A, Taborelli E, Corfield F, Schmidt U, Treasure J, Micali N (2013). Recognising the symptoms: how common are eating disorders in pregnancy?. Eur Eat Disord Rev.

[28] Knoph Berg C, Torgersen L, Von Holle A, Hamer RM, Bulik CM, Reichborn-Kjennerud T (2011). Factors associated with binge eating disorder in pregnancy. Int J Eat Disord.

[29] Lin HY, Hunag CK, Tai CM, Lin HY, Kao YH, Tsai CC, Hsuan CF, Lee SL, Chi SC, Yen YC (2013). Psychiatric disorders of patients seeking obesity treatment. BMC Psychiatry.

[30] Lundgren JD, Rempfer MV, Brown CE, Goetz J, Hamera E (2010). The prevalence of night eating syndrome and binge eating disorder among overweight and obese individuals with serious mental illness. Psychiatry Res.

[31] Machado PP, Goncalves S, Hoek HW (2013). DSM-5 reduces the proportion of EDNOS cases: evidence from community samples. Int J Eat Disord.

[32] Meltzer-Brody S, Zerwas S, Leserman J, Holle AV, Regis T, Bulik C (2011). Eating disorders and trauma history in women with perinatal depression. J Womens Health (Larchmt).

[33] Mousa TY, Al-Domi HA, Mashal RH, Jibril MAK (2010). Eating disturbances among adolescent schoolgirls in Jordan. Appetite.

[34] Ricca V, Castellini G, Lo Sauro C, Ravaldi C, Lapi F, Mannucci E, Rotella CM, Faravelli C (2009). Correlations between binge eating and emotional eating in a sample of overweight subjects. Appetite.

[35] Saka M, Türker FP, Bas M, Metin S, Yilmaz B, Köseler E (2012). An examination of food craving and eating behaviour with regard to eating disorders among adolescent. HealthMED.

[36] Sallet PC, de Alvarenga PG, Ferrao Y, de Mathis MA, Torres AR, Marques A, Hounie AG, Fossaluza V, do Rosario MC, Fontenelle LF, Petribu K, Fleitlich-Bilyk B (2010). Eating disorders in patients with obsessive-compulsive disorder: prevalence and clinical correlates. Int J Eat Disord.

[37] Tong J, Miao S, Wang J, Yang F, Lai H, Zhang C, Zhang Y, Hsu LK (2014). A two-stage epidemiologic study on prevalence of eating disorders in female university students in Wuhan, China. Soc Psychiatry Psychiatr Epidemiol.

[38] Zahodne LB, Susatia F, Bowers D, Ong TL, Jacobson CE, Okun MS, Rodriguez RL, Malaty IA, Foote KD, Fernandez HH (2011). Binge eating in Parkinson’s disease: prevalence, correlates and the contribution of deep brain stimulation. J Neuropsychiatry Clin Neurosci.

[39] Dahl JK, Eriksen L, Vedul-Kjelsas E, Strommen M, Kulseng B, Marvik R, Holan A (2010). Prevalence of all relevant eating disorders in patients waiting for bariatric surgery: a comparison between patients with and without eating disorders. Eat Weight Disord.

[40] McElroy SL, Frye MA, Hellemann G, Altshuler L, Leverich GS, Suppes T, Keck PE, Nolen WA, Kupka R, Post RM (2011). Prevalence and correlates of eating disorders in 875 patients with bipolar disorder. J Affect Disord.

[41] Swanson SA, Saito N, Borges G, Benjet C, Aguilar-Gaxiola S, Medina-Mora ME, Breslau J (2012). Change in binge eating and binge eating disorder associated with migration from Mexico to the U.S. J Psychiatr Res.

[42] Trace SE, Thornton LM, Root TL, Mazzeo SE, Lichtenstein P, Pedersen NL, Bulik CM (2012). Effects of reducing the frequency and duration criteria for binge eating on lifetime prevalence of bulimia nervosa and binge eating disorder: implications for DSM-5. Int J Eat Disord.

[43] Grenon R, Tasca GA, Cwinn E, Coyle D, Sumner A, Gick M, Bissada H (2010). Depressive symptoms are associated with medication use and lower health-related quality of life in overweight women with binge eating disorder. Womens Health Issues.

[44] Carano A, De Berardis D, Campanella D, Serroni N, Ferri F, Di Iorio G, Acciavatti T, Mancini L, Mariani G, Martinotti G, Moschetta FS, Di Giannantonio M (2012). Alexithymia and suicide ideation in a sample of patients with binge eating disorder. J Psychiatr Pract.

[45] De Zwaan M, Lancaster KL, Mitchell JE, Howell LM, Monson N, Roerig JL, Crosby RD (2002). Health-related quality of life in morbidly obese patients: effect of gastric bypass surgery. Obes Surg.

[46] De Zwaan M, Mitchell JE, Howell LM, Monson N, Swan-Kremeier L, Roerig JL, Kolotkin RL, Crosby RD (2002). Two measures of health-related quality of life in morbid obesity. Obes Res.

[47] Doll HA, Petersen SE, Stewart-Brown SL (2005). Eating disorders and emotional and physical well-being: associations between student self-reports of eating disorders and quality of life as measured by the SF-36. Qual Life Res.

[48] Masheb RM, Grilo CM (2009). Quality of life in patients with binge eating disorder. Eat Weight Disord.

[49] Mond JM, Hay PJ, Rodgers B, Owen C, Beumont PJ (2005). Assessing quality of life in eating disorder patients. Qual Life Res.

[50] Padierna A, Quintana JM, Arostegui I, Gonzalez N, Horcajo MJ (2000). The health-related quality of life in eating disorders. Qual Life Res.

[51] Rieger E, Wilfley DE, Stein RI, Marino V, Crow SJ (2005). A comparison of quality of life in obese individuals with and without binge eating disorder. Int J Eat Disord.

[52] Kolotkin RL, Westman EC, Ostbye T, Crosby RD, Eisenson HJ, Binks M (2004). Does binge eating disorder impact weight-related quality of life?. Obes Res.

[53] Dickerson JF, DeBar L, Perrin NA, Lynch F, Wilson GT, Rosselli F, Kramer HC, StriegelMoore RH (2011). Health-service use in women with binge eating disorders. Int J Eat Disord.

[54] Mond JM, Hay PJ, Rodgers B, Owen C (2007). Health service utilization for eating disorders: findings from a community-based study. Int J Eat Disord.

[55] Striegel-Moore RH, Dohm FA, Wilfley DE, Pike KM, Bray NL, Kraemer HC, Fairburn CG (2004). Toward an understanding of health services use in women with binge eating disorder. Obes Res.

