# Supplementary material for: Epidemiology, health-related quality of life and economic burden of binge eating disorder: a systematic literature review
Source: Eat Weight Disord. 2015 Jan 9;20(1):1–12. doi: 10.1007/s40519-014-0173-9 (PMC4349998; doi:10.1007/s40519-014-0173-9)
Supplement: Supplementary file 1 — Supplementary material 1 (PDF 277 kb) [file 40519_2014_173_MOESM1_ESM.pdf]

**Online Resource 1** Search strategy used in the systematic literature review

**Article title**: Epidemiology, Health-Related Quality of Life and Economic Burden of Binge Eating Disorder: a Systematic Literature Review

**Journal name**: Eating and Weight Disorders

**Authors**: Tamás Ágh[1], Gábor Kovács[1], Manjiri Pawaskar[2] , Dylan Supina[2], András Inotai[1], Zoltán Vokó[1,3]

1. Syreon Research Institute, Budapest, Hungary
2. Shire Development LLC., Wayne, PA, USA
3. Eötvös Loránd University, Department of Health Policy and Health Economics, Budapest, Hungary

**Corresponding author**:

Tamás Ágh

Syreon Research Institute, Thököly Street 119., 1146 Budapest, Hungary

E-mail: tamas.agh@syreon.eu

Search strategy used in the systematic literature review

| Domain | Sub-category | Search | Search terms: Medline and Embase (via Scopus) | Search terms: PsycINFO, PsycARTICLES, Academic Search Complete, CINAHL Plus , Business Source Premier (via Ebsco Host) and Cochrane Library (includes Cochrane Database of Systematic Reviews, Cochrane Central Register of Controlled Trials, Cochran Methodology Register, Database of Abstracts of Reviews of Effects, Health Technology Assessment Database and NHS Economic Evaluation Database) |
|---|---|---|---|---|
| **Disease** | **Binge Eating Disorder / Bulimia Nervosa / Anorexia Nervosa / Eating disorder not otherwise specified** | 1 | "eating disorder" OR "binge eating disorder" OR (eat* W/2 disord*) OR "bulimia nervosa" OR bulimia OR bulimi* OR purg* OR bing* OR overeat* OR (compulsive W/2 eat*) OR (compulsive W/2 vomit*) OR (food* AND bing*) OR (self?induc* W/2 vomit*) OR "anorexia nervosa" OR (anorex* AND nervosa) | "eating disorder" OR "binge eating disorder" OR "bulimia nervosa" OR overeating OR "compulsive eating" OR "compulsive vomiting" OR purging OR "self-induced vomiting" OR "anorexia nervosa" |
| **Epidemiology** | **Prevalence / Incidence / Mortality** | 2 | prevalence OR incidence OR mortality | prevalence OR incidence OR mortality |
| **Health-related quality of life burden** | **Quality of life** | 3 | "quality of life" OR QoL | "quality of life" OR QoL |
| | **Health burden / Humanistic burden / Quality adjusted life years / Disability adjusted life years** | 4 | burden OR (burden w/3 disease) OR (burden w/3 illness) OR (humanistic w/2 burden) OR (clinic* w/2 burden) OR (life w/4 years w/3 lost) OR (premat* mortal*) OR "quality adjusted life year*" OR QALY* OR "disability adjusted life year*" OR DALY* | "burden" OR "burden of disease" OR "burden of illness" OR "humanistic burden" OR "clinical burden" OR "life years lost" OR "premature mortality" OR "quality adjusted life years" OR "QALY" OR "disability adjusted life years" OR "DALY" |
| **Economic burden** | **Direct healthcare cost** | 5 | "economic*" OR "cost*" OR "resource*" OR "2esourc*" OR (econ* W/2 burden) OR (econ* W/2 analys*) OR (econ* W/2 eval*) OR (econ* W/2 study) OR (econ* W/2 assess*) OR (econ* W/2 conseq*) OR (cost* W/3 ill*) OR (cost* W/2 health*) OR (cost* W/2 burden) OR (cost* W/3 illness) OR (cost* W/3 disease) OR (cost* W/2 analys*) OR (cost* W/2 assess*) OR (cost* W/2 study) OR (2esource* W/2 use) OR (health* W/2 resource*) OR (resource* W/2 utili*) OR expendit* OR (financial W/2 burden) | "economic burden" OR "cost burden" OR "resource burden" OR "financial burden" OR "economic consequences" OR "cost of illness" OR "healthcare cost" OR "cost of disease" OR "cost analysis" OR "cost assessment" OR "cost study" OR "resource use" OR "healthcare resources" OR "resource utilization" OR "expenditure" |
| | **Direct patient and caregiver cost** | 6 | (out w/3 pocket) or (patient w/3 cost*) or (co?pay*) or (privat* w/2 expendit*) or (patient w/3 time) or (carer* w/3 cost) or (carer* w/2 expendit*) or (carer* w/3 time) or (caregiv* w/3 cost) or (caregiv* w/2 expendit*) or (caregiv* w/3 time) | "out of pocket" OR "patient cost" OR "co-payment" OR "private expenditure" OR "patient time" OR "carer cost" OR "carer expenditure" OR "carer time" OR "caregiver cost" OR "caregiver expenditure" OR "caregiver time" |
| | **Wider societal (and intangible) cost** | 7 | (cost* w/4 societ*) or (cost* w3 social*) or (cost w/3 social* w/3 care*) or (work?loss) or (work* w/3 absent*) or (product* w/3 loss*) or (lost* w/2 product*) or (human capital) or (cost* w/2 friction) or earning* or (educ* w/3 attainm*) or (educ* w/3 achiev*) or (educ* w/3 impairm*) or (occup* w/3 attainm*) or (occup* w/3 achiev*) or (occup* w/3 impairm*) or (social* w.3 funct*) or (social* w.3 impairm*) or (caregiv* w/2 burden) or (carer* w/2 burden) or (famil*w/2 burden) | "societal cost" OR "social cost" OR "social care cost" OR "work loss" OR "work absenteeism" OR "productivity loss" OR "lost productivity" OR "earnings" OR "educational attainment" OR "educational achievement" OR "educational impairment" OR "occupational attainment" OR "occupational achievement" OR "occupational impairment" OR "social functioning" OR "social impairment" OR "caregiver burden" OR "carer burden" OR "family burden" |
| **Combined search in titles, abstracts and keywords** | | | #1 AND (#2 OR #3 OR #4 OR #5 OR #6 OR #7) | |

Search was performed in July 2013.

No limits were defined in terms of publication date in case of search #1, #3, #4, #5, #6 and #7. Search #2 was limited to 2009 and 2013
