# Supplementary material for: Epidemiology, health-related quality of life and economic burden of binge eating disorder: a systematic literature review
Source: Eat Weight Disord. 2015 Jan 9;20(1):1–12. doi: 10.1007/s40519-014-0173-9 (PMC4349998; doi:10.1007/s40519-014-0173-9)
Supplement: Supplementary file 2 — Supplementary material 2 (PDF 397 kb) [file 40519_2014_173_MOESM2_ESM.pdf]

Online Resource 2 The general characteristics of the included studies

Article title: Epidemiology, Health-Related Quality of Life and Economic Burden of Binge Eating Disorder: a Systematic Literature Review

Journal name: Eating and Weight Disorders

Authors: Tamás Ágh[1], Gábor Kovács[1], Manjiri Pawaskar[2] , Dylan Supina[2], András Inotai[1], Zoltán Vokó[1,3]

1. Syreon Research Institute, Budapest, Hungary
2. Shire Development LLC., Wayne, PA, USA
3. Eötvös Loránd University, Department of Health Policy and Health Economics, Budapest, Hungary

Corresponding author:

Tamás Ágh

Syreon Research Institute, Thököly Street 119., 1146 Budapest, Hungary

E-mail: tamas.agh@syreon.eu

The general characteristics of the included studies

First author, publication year	Study characteristic						Sample characteristic					Prevalence of BED		
	**Country**	**Study design**	**Study year**	**Included eating disorders**	**Diagnostic criteria of BED**	**Diagnostic method of BED**	**Sample**	**Sample size**	**Sample age** range/ mean(SD)	**Percentage of female**	**BMI** range/ mean(SD)	**Point**	**12-month**	**Lifetime**
Ackard, 2011 [22]	US	CS	1998-1999	BN, BED	DSM-IV	-	BED<br>TOTAL	53<br>4,746	-<br>14.9(1.7)	85%<br>50%	-<br>-	1.1%* (female: 1.2%*, male: 0.3%*)	n.a.	n.a.
Anderson, 2012 [23]	US	CS	2008	BN, BED	DSM-IV	Q-EDD	BED<br>TOTAL (gymnasts, swimmers)	2<br>414	-<br>19.1(1.9)	100%<br>100%	-<br>-	0.5%*	n.a.	n.a.
Azarbad, 2010 [24]	US	CS	2002-2009	BED	DSM-IV	-	BED<br>TOTAL	-<br>404	-<br>41.5(10.4)	-<br>100%	-<br>50.6(9.3)	Caucasian: 15.7%<br>African American: 11.5%<br>Hispanic: 11.8%	n.a.	n.a.
Bedrosian, 2011 [25]	US	CS	2008-2009	BN, BED	DSM-IV	EDE-Q	BED<br>TOTAL	559<br>4,051	 40.7(10.6)	81%<br>75%	female: 34.6(8.5) male: 41.6(11.1)<br>female 32.2(7.8) male 31.6(6.1)	13.8%* (female: 14.7%, male: 10.9%)	n.a.	n.a.
Canan, 2011 [20]	Turkey	CS	-	BED	DSM-IV	SCID	BED<br>TOTAL (individuals with type 2 diabetes)	27<br>82	48.5(8.4)<br>49.8(10.2)	67%<br>66%	30.6(5.5)<br>30.3(5.5)	34.1%	n.a.	n.a.
Carano, 2012 [44]	Italy	CS	-	BED	DSM-IV	SCID	BED	80	39.5(8.2)	53%	36.2(7.7)	n.a.	n.a.	n.a.
Cassin, 2008 [10]	Canada	LS (16 weeks)	2004-2005	BED	DSM-IV	SCID	BED	108	42.5 (12.7)	100%	33.2(7.8)	n.a.	n.a.	n.a.
Czarlinski, 2012 [26]	US	CS	-	AN, BN, BED, EDNOS	DSM-IV	SCID	BED<br>TOTAL (residents of Oxford House: provide an abstinent living environment)	-<br>31	-<br>41.9(9.46)	-<br>100%	-<br>28.79(7.44)	16.1%	n.a.	n.a.
Dahl, 2010 [39]	Norway	CS	2005	BN, BED, EDNOS	DSM-IV	EDO	BED<br>TOTAL (obese individuals)	21<br>157	40.3(9.0)<br>41.0(10.4)	76%<br>73%	46.8(6.2)<br>47.1(5.7)	13.4% (female: 13.9%*, male: 11.9%*)	n.a.	n.a.

First author, publication year	Study characteristic						Sample characteristic					Prevalence of BED		
	**Country**	**Study design**	**Study year**	**Included eating disorders**	**Diagnostic criteria of BED**	**Diagnostic method of BED**	**Sample**	**Sample size**	**Sample age** range/ mean(SD)	**Percentage of female**	**BMI** range/ mean(SD)	**Point**	**12-month**	**Lifetime**
De Zwaan, 2002 [45]	US	CS	-	BED	DSM-IV	QEWP-R	BED	38 (before surgery) / 9 (after surgery)	-	-	-	not published between 2009 and 2013		
							TOTAL (individuals before/after gastric bypass surgery)	78	-	83%	-			
De Zwaan, 2002 [46]	US	CS	1999-2001	BED	DSM-IV	QEWP-R	BED	19	41.3(9.0)	100%	48.0(7.9)	not published between 2009 and 2013		
							TOTAL (obese individuals)	110	19-62/39.6	87%	35.4-86.9/48.4			
Dickerson, 2011 [53]	US	CS	-	BED, RBE	DSM-IV	EDE	BED	50	36.4(7.3)	100%	33.4(6.1)	n.a.	n.a.	n.a.
							TOTAL	100		100%				
Doll, 2005 [47]	UK	CS	-	AN, BN, BED	DSM-IV		BED	22	-	86%	-	not published between 2009 and 2013		
							TOTAL	1,439	23.4(6.8)	63%	22.6(3.4)			
Easter, 2013 [27]	UK	CS	2010-2012	AN, BN, BED, EDNOS	DSM-IV	EDDS	BED	13	-	-	-	6-12 months prior the pregnancy: 0.1%<br>during pregnancy: 1.8%	n.a.	n.a.
							TOTAL (pregnants)	739	30.5(5.8)	100%	23.8(4.7)			
Faulconbridge, 2013 [11]	US	LS (12 months)	-	BED	proposed DSM-5	EDE	BED, bariatric surgery (obese individuals)	36	47.0(1.6)	73%	48.9(1.1)	n.a.	n.a.	n.a.
							BED, lifestyle intervention (obese individuals)	49	43.8(1.4)	80%	44.3(0.7)			
Grenon, 2010 [43]	Canada	CS	-	BED	DSM-IV	SCID	BED (obese individuals)	105	44.3(11.8)	100%	38.2(6.8)	n.a.	n.a.	n.a.
Hsu, 2002 [19]	US	CS	-	BED	DSM-IV	EDE	BED	4	-	-	-	not published between 2009 and 2013		
							TOTAL (obese individuals)	37	-	84%	-			
Hudson, 2012 [21]	US	CS	-	BED	DSM-IV	-	BED	-	-	-	-	DSM-IV: female: 1.6%, male: 0.8%<br>DSM-5: female: 1.7%, male: 0.8%	n.a.	DSM-IV: female: 3.5%, male: 2.0%<br>DSM-5: female: 3.6%, male: 2.1%
					proposed DSM-5		TOTAL (first-degree relatives of BED patients)	888	46.7(17.4)	66%	28.7			
Kessler, 2013 [2]	Colombia, Brazil, Mexico, Romania, Belgium, France, Germany, Italy, The Netherlands, New Zeeland, Northern Ireland, Portugal, Spain, US	CS	-	BN, BED	DSM-IV	WHO CIDI	BED	-	-	-	-	n.a.	0.8%	1.9%
							TOTAL	24,124	18+	-	-			

First author, publication year	Study characteristic						Sample characteristic					Prevalence of BED		
	Country	Study design	Study year	Included eating disorders	Diagnostic criteria of BED	Diagnostic method of BED	Sample	Sample size	Sample age range/ mean(SD)	Percentage of female	BMI range/ mean(SD)	Point	12-month	Lifetime
Knoph, 2013 [12]	Norway	LS (36 months)	1998-2008	AN, BN, BED, EDNOS	DSM-IV	-	BED<br>TOTAL (women in postpartum period)	-<br>77,807	-<br>-	-<br>100%	-<br>-	before pregnancy: 3.0%<br>during pregnancy: 4.3%<br>18 months postpartum: 2.7%<br>36 months postpartum: 3.1%	n.a.	n.a.
Knoph Berg, 2011 [28]	Norway	CS	1999-2009 (data 2007)	BED	DSM-IV	-	BED<br>TOTAL (pregnants)	1,887<br>45,644	-<br>29.9	-<br>100%	-<br>-	4.1%	n.a.	n.a.
Kolotkin, 2004 [52]	US	CS	-	BED	DSM-IV	QEWP-R	BED<br>TOTAL	95<br>530	45.0(12.6)<br>48.9(14.1)	75.8%<br>59.8%	42.0(10.4)<br>39.1(10.4)	not published between 2009 and 2013		
Lin, 2013 [29]	China	CS	2007-2010	AN, BN, BED	DSM-IV	SCID	BED<br>TOTAL (individuals seeking obesity treatment)	64<br>841	-<br>35.5(11.6)	89%<br>69%	-<br>35.7(8.9)	7.6% (female: 9.8%, male: 2.7%)	n.a.	n.a.
Lundgren, 2010 [30]	US	CS	-	BED, NES	DSM-IV	EDE	BED<br>TOTAL (obese individuals with serious mental illness)	4<br>68	-<br>43.9(10.4)	-<br>60%	-<br>37.2(8.1)	5.9%	n.a.	n.a.
Machado, 2013 [31]	Portugal	CS	-	AN, BN, BED, EDNOS	proposed DSM-5	EDE-Q, EDE	BED<br>TOTAL	19<br>3,048	20.2(4.9)	100%<br>100%	20.2(2.8)	0.62%	n.a.	n.a.
Marques, 2011 [13]	US	retros-pective data analysis	-	AN, BN, BED	DSM-IV	WHO-CIDI	-	-	-	-	-	n.a.	Latino: 1.1%<br>Non-latinos White: 0.6%<br>African American: 0.7%<br>Asian:0.7%	Latino: 2.1%<br>Non-latinos White: 1.4%<br>African American: 1.5%<br>Asian: 1.2%
Masheb, 2004 [48]	US	CS	-	BED	DSM-IV	SCID	BED	94	44.9(8.3)	78%	35.2(8.1)	n.a.	n.a.	n.a.
McElroy, 2011 [40]	US	CS	-	AN, BN, BED	DSM-IV	SCID	BED<br>TOTAL (outpatients with bipolar I and II disorder)	77<br>875	40.6(10.3)<br>-	76%<br>-	-<br>-	n.a.	n.a.	8.8%
Meltzer-Brody, 2011 [32]	US	CS	2006-2009	AN, BN, BED, EDNOS	DSM-IV	SCID	BED<br>TOTAL (women with perinatal depression)	11<br>158	31.8(6.4)<br>30.2(5.9)	100%<br>100%	-<br>-	7.0%	n.a.	n.a.

First author, publication year	Study characteristic						Sample characteristic					Prevalence of BED		
	**Country**	**Study design**	**Study year**	**Included eating disorders**	**Diagnostic criteria of BED**	**Diagnostic method of BED**	**Sample**	**Sample size**	**Sample age** range/ mean(SD)	**Percentage of female**	**BMI** range/ mean(SD)	**Point**	**12-month**	**Lifetime**
Mond, 2005 [49]	Australia	CS	2001-2002	AN, BN, BED	DSM-IV	EDE-Q	BED<br>Control	10<br>495	34.3(7.4)<br>33.5(8.9)	100%<br>100%	41.2(11.7)<br>24.9(6.5)	n.a.	n.a.	n.a.
Mond, 2007 [54]	Australia	CS	-	AN, BN, BED, EDNOS	DSM-IV	EDE-Q	BED<br>Control	20<br>5,096	-<br>30.3(7.2)	-<br>-	-<br>24.4(5.2)	n.a.	n.a.	n.a.
Mousa, 2010 [33]	Jordan	CS	2008	AN, BN, BED, EDNOS	DSM-IV	EAT-26	BED<br>TOTAL	6<br>326	-<br>12.9(1.8)	100%<br>100%	-<br>20.9(3.8)	1.8%	n.a.	n.a.
Padierna, 2000 [51]	Spain	CS	-	AN, BN, BED	DSM-IV	-	BED<br>TOTAL	17<br>197	-<br>23.4(6.7)	-<br>-	-<br>-	n.a.	n.a.	n.a.
Perez, 2012 [14]	US	retros-pective data analysis	-	BED	DSM-IV	CIDI	BED<br>TOTAL	non-obese: 124<br>obese: 126<br>16,898	-	-<br>57%	nonobese: 25.3(3.2)<br>obese: 36.3(5.2)<br>-	1.5%*	n.a.	n.a.
Preti, 2009 [3]	Belgium, France, Germany, Italy, The Netherlands, Spain	CS	2001-2003	AN, BN, BED	DSM-IV	CIDI	BED<br>TOTAL	-<br>21,425	-<br>18+	-<br>52%	-<br>-	n.a.	0.1% (female:0.6%, male: 0.1%)	1.1% (female: 1.9%, male: 0.3%)
Ricca, 2009 [34]	Italy	CS	2004-2007	BED	DSM-IV	SCID	BED<br>TOTAL (obese individuals seeking weight loss treatment)	105<br>438	44.9(12.7)<br>46.6(13.4)	87%<br>83%	38.1(7.4)<br>-	23.9%	n.a.	n.a.
Rieger, 2005 [51]	US	CS	-	BED	DSM-IV	EDE-Q	BED<br>TOTAL (obese individuals)	56<br>118	42(10.0)<br>-	89%<br>-	36.6(5.1)<br>-	n.a.	n.a.	n.a.
Saka, 2012 [35]	Turkey	CS	-	BN, BED, NES	DSM-IV	QEWP-R	BED<br>TOTAL	22<br>563		77%<br>67%	27.6(5.3)<br>-	3.9% (female: 4.5%, male: 2.7%)	n.a.	n.a.
Sallett, 2010 [36]	Brazil	CS	2003-2008	AN, BN, BED	DSM-IV	SCIDI	BED<br>TOTAL (individuals with obsessive-compulsive disorder)	37<br>815		57%<br>58%	-<br>-	4.5% (female: 4.4%; male: 4.7%)	n.a.	7.9% (female: 8.6%, male: 6.8%)
Silveria, 2005 [15]	Brazil	LS (12 week)	-	BED	DSM-IV	-	BED	9	33.3(10.8)	100%	36.5(4.5)	n.a.	n.a.	n.a.
Stice, 2013 [16]	US	LS (8 years)	-	AN, BN, BED, FED-NEC	proposed DSM-5	EDDI	BED<br>TOTAL	-<br>496	-<br>13 (baseline)	-<br>100%	-<br>-	n.a.	n.a.	3.0% (by age 20)

First author, publication year	Study characteristic						Sample characteristic					Prevalence of BED		
	Country	Study design	Study year	Included eating disorders	Diagnostic criteria of BED	Diagnostic method of BED	Sample	Sample size	Sample age range/ mean(SD)	Percentage of female	BMI range/ mean(SD)	Point	12-month	Lifetime
Striegel-Moore, 2004 [55]	US	CS	-	BED	DSM-IV	SCID	BED<br>TOTAL	162<br>518	-<br>White: 30.2(5.8) Black: 29.7(5.9)	100%<br>100%	-<br>-	n.a.	n.a.	n.a.
Swanson, 2011 [4]	US	CS	-	AN, BN, BED	DSM-IV	CIDI	BED<br>TOTAL	-<br>10,123	-<br>13-18	-<br>-	-<br>-	n.a.	0.9% (female: 1.4%, male: 0.4%)	1.6% (female: 2.3%, male: 0.8%)
Swanson, 2012 [41]	US	CS	2001-2002	BED	DSM-IV	CIDI	BED<br>TOTAL	60<br>2,268	-<br>-	-<br>59%	-<br>-	n.a.	n.a.	in Mexico: 1.6%<br>in the US among Mexican-American: 2.2%
Tong, 2014 [37]	China	CS	2009	AN, BN, BED	DSM-IV	EDE, SCID	BED<br>TOTAL	SCID:48; EDE:56<br>8,444	-<br>-	-<br>100%	-<br>-	3.53%	n.a.	n.a.
Trace, 2012 [42]	Sweden	CS	2005	BN, BED	DSM-IV proposed DSM-5	SCID	BED<br>TOTAL (twins)	-<br>13,295	-<br>20-47	-<br>100%	-<br>-	n.a.	n.a.	DSM-IV: 0.17%<br>DSM-5: 0.2%
White, 2011 [17]	US	LS	1995, 2002, 2008	BN, BED, EDNOS	DSM-IV	WMQ	BED<br>TOTAL	-<br>1995: 493 2002: 272 2008: 641	-<br>1995: 21.1 2002: 20.9 2008: 20.4	-<br>1995: 69% 2002: 70% 2008: 69%	-<br>-	n.a.	n.a.	1995: 4.2%, 2002:1.5%, 2008:2.3% (female: 1995:5.6%, 2002:2.1%, 2008:3.0% male: 1995:1.2%, 2002:0.0%, 2008:1.0%)
Wilfley, 2008 [18]	-	LS (24 weeks)	-	BED	DSM-IV	EDE	BED, sibutramine<br>BED, placebo	152<br>152	41.8(9.7)<br>42.1(9.9)	90%<br>90%	35.5(5.8)<br>36.3(5.5)	n.a.	n.a.	n.a.
Zahodne, 2011 [38]	US	CS	2008	BED	DSM-IV	EDE-Q, EDDS	BED<br>TOTAL (individuals with Parkinson disease)	1<br>96	-<br>-	-<br>-	-<br>-	1.0%	n.a.	n.a.

AN: anorexia nervosa; BED: binge eating disorder; BN: bulimia nervosa; BIM: body mass index; CS: cross-sectional study; DSM-IV/5: 4th/5th Edition of The Diagnostic and Statistical Manual of Mental Disorders; EDDI: Eating Disorder Diagnostic Interview; EDDS: Eating Disorder Diagnostic Scale; EDE: Eating Disorder Examination; EDE-Q: Eating Disorder Examination Questionnaire; EDNOS: eating disorder otherwise not specified; EDO: Eating Disorders in Obesity; FED-NEC: feeding or eating disorders not elsewhere classified; LS: longitudinal study; n.a.: not applicable; NES: night eating syndrome; Q-EDD: 50-item Questionnaire for Eating Disorder Diagnoses; QEWP-R: Questionnaire on Eating and Weight Patterns; SCID: Structured Clinical Interview for DSM Disorder; SD: standard deviation; SIED: Screening Interview for Eating Disorders; UK: United Kingdom; US: United States; WHO CIDI: World Health Organization Composite International Diagnostic Interview; WMQ: Weight Management Questionnaire

*: only the number of BED patients were reported, prevalence rate was calculated subsequently based on the total sample size and the number of BED patients
