# Supplementary material for: Epidemiology, health-related quality of life and economic burden of binge eating disorder: a systematic literature review
Source: Eat Weight Disord. 2015 Jan 9;20(1):1–12. doi: 10.1007/s40519-014-0173-9 (PMC4349998; doi:10.1007/s40519-014-0173-9)
Supplement: Supplementary file 3 — Supplementary material 3 (PDF 308 kb) [file 40519_2014_173_MOESM3_ESM.pdf]

Online Resource 3 Quality assessment of the included studies

Article title: Epidemiology, Health-Related Quality of Life and Economic Burden of Binge Eating Disorder: a Systematic Literature Review

Journal name: Eating and Weight Disorders

Authors: Tamás Ágh[1], Gábor Kovács[1], Manjiri Pawaskar[2] , Dylan Supina[2], András Inotai[1], Zoltán Vokó[1,3]

1. Syreon Research Institute, Budapest, Hungary
2. Shire Development LLC., Wayne, PA, USA
3. Eötvös Loránd University, Department of Health Policy and Health Economics, Budapest, Hungary

Corresponding author:

Tamás Ágh

Syreon Research Institute, Thököly Street 119., 1146 Budapest, Hungary

E-mail: tamas.agh@syreon.eu

Quality assessment of the included studies

Item (item number) / First author, publication year		Ackard, 2011 [22]	Anderson, 2012 [23]	Azarbad, 2010 [24]	Bedrosian, 2011 [25]	Canan, 2011 [20]	Carano, 2012 [44]	Cassin, 2008 [10]	Czarlinski, 2012 [26]	Dahl, 2010 [39]	De Zwaan, 2002 [45]	De Zwaan, 2002 [46]	Dickerson, 2011 [53]
**Title and abstract**	(1.a)	✓	✓	✓	✓	✓	✓	✓	✓	✓	✓	✓	✓
	(1.b)	✓	-	✓	✓	✓	✓	✓	✓	✓	✓	✓	✓
**Introduction**	Background/rationale (2)	✓	✓	✓	✓	-	✓	✓	✓	✓	✓	✓	✓
	Objectives (3)	✓	✓	✓	✓	?	✓	✓	✓	✓	?	?	✓
	Study design (4)	✓	?	-	?	✓	?	✓	✓	✓	✓	✓	✓
	Setting (5)	✓	✓	✓	✓	?	✓	✓	✓	✓	?	✓	?
	Participants (6.a)	✓	?	?	✓	?	✓	✓	?	✓	✓	✓	✓
	Participants (6.b)	n.a.	n.a.	n.a.	n.a.	n.a.	n.a.	✓	n.a.	n.a.	✓	✓	?
	Variables (7)	?	?	✓	?	✓	✓	✓	✓	✓	✓	✓	✓
	Data sources/measurement (8)	✓	✓	✓	?	✓	✓	✓	✓	✓	✓	✓	✓
**Methods**	Bias (9)	✓	-	✓	-	-	-	?	✓	-	-	?	-
	Study size (10)	✓	✓	✓	✓	?	✓	✓	?	✓	✓	✓	✓
	Quantitative variables (11)	✓	✓	✓	✓	✓	✓	✓	✓	✓	✓	✓	✓
	Statistical methods (12.a)	✓	?	✓	✓	?	✓	✓	✓	✓	✓	✓	✓
	Statistical methods (12.b)	✓	-	✓	✓	-	✓	-	✓	✓	✓	-	✓
	Statistical methods (12.c)	-	?	✓	✓	-	-	?	-	✓	-	✓	-
	Statistical methods (12.d)	?	✓	✓	✓	✓	?	✓	?	✓	✓	✓	-
	Statistical methods (12.e)	-	-	-	-	-	-	-	-	-	-	-	-
	Participants (13.a)	?	✓	✓	✓	?	✓	✓	✓	✓	✓	✓	✓
	Participants (13.b)	?	-	✓	✓	-	?	✓	-	✓	-	?	-
	Participants (13.c)	-	-	-	-	-	-	✓	-	-	-	-	-
	Descriptive data (14.a)	✓	✓	✓	✓	?	?	✓	?	✓	✓	✓	✓
	Descriptive data (14.b)	-	-	-	?	-	-	?	-	✓	-	-	✓
**Results**	Descriptive data (14.c)	n.a.	n.a.	n.a.	n.a.	n.a.	n.a.	✓	n.a.	n.a.	n.a.	✓	?
	Outcome data (15)	✓	✓	✓	✓	✓	✓	✓	✓	✓	✓	✓	✓
	Main results (16.a)	✓	✓	✓	✓	✓	✓	✓	✓	✓	✓	✓	✓
	Main results (16.b)	✓	?	✓	✓	-	✓	✓	✓	✓	✓	✓	✓
	Main results (16.c)	n.a.	n.a.	n.a.	?	n.a.	n.a.	-	n.a.	?	n.a.	n.a.	n.a.
	Other analyses (17)	?	-	✓	✓	-	?	-	?	✓	-	✓	✓
	Key results (18)	✓	✓	✓	✓	✓	✓	✓	✓	✓	✓	✓	✓
**Discussion**	Limitations (19)	✓	✓	✓	✓	?	✓	✓	✓	✓	✓	✓	✓
	Interpretation (20)	✓	✓	✓	✓	✓	✓	✓	✓	✓	?	✓	✓
	Generalisability (21)	✓	✓	✓	✓	?	-	✓	✓	?	-	✓	✓
**Other information**	Funding (22)	-	?	-	-	-	-	-	-	-	-	✓	-
**Rate of criteria each study fulfilled (%)**		67.7%	51.6%	80.6%	71.9%	35.5%	61.3%	76.5%	64.5%	81.3%	62.5%	78.8%	69.7%

Item (item number) / First author, publication year		Doll, 2005 [47]	Easter, 2013 [27]	Faulconbridge, 2013 [11]	Grenon, 2010 [43]	Hsu, 2002 [19]	Hudson, 2012 [21]	Kessler, 2013 [2]	Knoph, 2013 [12]	Knoph Berg, 2011 [28]	Kolotkin, 2004 [52]	Lin, 2013 [29]	Lundgren, 2010 [30]
**Title and abstract**	(1.a)	✓	✓	✓	✓	✓	?	✓	✓	✓	✓	✓	✓
	(1.b)	✓	✓	✓	✓	✓	✓	✓	✓	✓	✓	✓	✓
**Introduction**	Background/rationale (2)	✓	✓	✓	✓	?	?	-	✓	✓	✓	✓	✓
	Objectives (3)	✓	?	✓	✓	?	✓	?	✓	-	✓	✓	?
	Study design (4)	?	?	?	-	✓	?	?	✓	✓	✓	✓	✓
	Setting (5)	✓	✓	✓	-	✓	?	✓	✓	✓	?	✓	✓
	Participants (6.a)	✓	?	✓	-	✓	✓	✓	✓	✓	✓	✓	✓
	Participants (6.b)	n.a.	n.a.	✓	n.a.	✓	n.a.	n.a.	?	?	n.a.	n.a.	n.a.
	Variables (7)	✓	✓	✓	✓	✓	-	✓	✓	✓	✓	?	✓
	Data sources/measurement (8)	✓	✓	✓	✓	✓	?	✓	✓	✓	✓	✓	✓
**Methods**	Bias (9)	✓	-	-	-	-	-	-	✓	-	-	-	?
	Study size (10)	✓	?	✓	?	✓	✓	✓	✓	✓	✓	✓	?
	Quantitative variables (11)	✓	?	✓	✓	✓	-	✓	✓	✓	✓	✓	✓
	Statistical methods (12.a)	✓	-	✓	✓	✓	-	✓	✓	✓	✓	✓	✓
	Statistical methods (12.b)	-	-	-	✓	-	-	✓	✓	✓	✓	?	?
	Statistical methods (12.c)	?	?	✓	✓	-	-	?	✓	✓	-	✓	-
	Statistical methods (12.d)	?	-	✓	-	✓	-	-	✓	?	-	✓	?
	Statistical methods (12.e)	-	-	-	-	-	-	-	-	-	-	-	-
	Participants (13.a)	✓	✓	✓	✓	✓	✓	✓	✓	✓	✓	✓	?
	Participants (13.b)	-	-	✓	✓	-	-	-	✓	✓	-	✓	-
	Participants (13.c)	✓	-	✓	-	-	-	-	-	-	-	✓	-
	Descriptive data (14.a)	✓	?	✓	✓	✓	-	✓	✓	?	✓	✓	✓
	Descriptive data (14.b)	-	-	✓	✓	-	-	-	✓	?	-	?	-
**Results**	Descriptive data (14.c)	n.a.	n.a.	✓	n.a.	✓	n.a.	n.a.	-	-	n.a.	n.a.	n.a.
	Outcome data (15)	✓	✓	✓	✓	✓	✓	✓	?	?	✓	✓	✓
	Main results (16.a)	✓	✓	✓	✓	✓	?	✓	✓	✓	✓	✓	✓
	Main results (16.b)	✓	-	✓	✓	✓	-	✓	✓	?	✓	✓	✓
	Main results (16.c)	n.a.	n.a.	?	n.a.	n.a.	n.a.	✓	✓	✓	n.a.	n.a.	n.a.
	Other analyses (17)	-	-	✓	✓	-	-	?	?	-	✓	-	-
	Key results (18)	✓	✓	✓	✓	✓	✓	✓	✓	✓	✓	✓	✓
**Discussion**	Limitations (19)	?	✓	✓	✓	✓	✓	✓	✓	✓	✓	✓	?
	Interpretation (20)	✓	✓	✓	✓	✓	✓	✓	✓	✓	✓	✓	✓
	Generalisability (21)	?	-	✓	✓	-	?	✓	?	-	✓	?	?
**Other information**	Funding (22)	✓	✓	-	-	✓	-	✓	-	✓	✓	✓	✓
**Rate of criteria each study fulfilled (%)**		67.7%	41.9%	82.4%	71.0%	66.7%	26.5%	65.6%	76.5%	61.8%	74.2%	77.4%	54.8%

✓: yes; -: no; ?: partially; n.a.: not applicable

Item (item number) / First author, publication year		Machado, 2013 [31]	Marques, 2011 [13]	Masheb, 2004 [48]	McElroy, 2011 [40]	Meltzer-Brody, 2011 [32]	Mond, 2005 [49]	Mond, 2007 [54]	Mousa, 2010 [33]	Padierna, 2000 [50]	Perez, 2012 [14]	Preti, 2009 [3]	Ricca, 2009 [34]	Rieger, 2005 [51]	Saka, 2012 [35]	Sallet, 2010 [36]	Silveira, 2005 [15]	Stice, 2013 [16]	Striegel-Moore, 2004 [55]	Swanson, 2011 [4]	Swanson, 2012 [41]	Tong, 2014 [37]	Trace, 2012 [42]	White, 2011 [17]	Wilfley, 2008 [18]	Zahodne, 2011 [38]
**Title and abstract**	(1.a)	✓	✓	✓	✓	✓	✓	✓	✓	✓	✓	✓	✓	✓	✓	✓	✓	✓	✓	✓	?	✓	✓	✓	✓	✓
	(1.b)	✓	?	✓	✓	✓	✓	✓	✓	✓	✓	✓	✓	✓	✓	✓	✓	✓	✓	✓	✓	✓	✓	✓	✓	?
**Introduction**	Background/rationale (2)	✓	✓	✓	✓	✓	✓	✓	✓	✓	✓	✓	✓	✓	✓	✓	✓	✓	✓	✓	✓	?	✓	✓	✓	?
	Objectives (3)	✓	✓	?	✓	✓	✓	✓	?	?	✓	✓	?	✓	?	✓	?	?	?	✓	✓	-	✓	✓	?	?
**Methods**	Study design (4)	✓	✓	?	✓	✓	✓	✓	✓	✓	✓	✓	✓	?	?	✓	✓	✓	✓	✓	✓	✓	?	✓	✓	✓
	Setting (5)	?	?	-	?	✓	✓	✓	✓	?	✓	✓	✓	✓	?	✓	✓	✓	✓	?	✓	✓	✓	✓	?	✓
	Participants (6.a)	✓	?	✓	✓	✓	✓	✓	✓	✓	?	✓	✓	✓	?	?	✓	✓	✓	?	✓	✓	✓	✓	✓	✓
	Participants (6.b)	n.a.	n.a.	?	n.a.	-	?	n.a.	n.a.	n.a.	n.a.	n.a.	n.a.	n.a.	n.a.	n.a.	n.a.	n.a.	n.a.	n.a.	n.a.	✓	n.a.	n.a.	✓	n.a.
	Variables (7)	✓	✓	✓	✓	✓	✓	✓	✓	✓	✓	✓	✓	✓	✓	✓	✓	✓	✓	✓	✓	✓	✓	✓	✓	✓
	Data sources/measurement (8)	✓	✓	✓	?	✓	✓	✓	✓	✓	✓	✓	✓	✓	✓	✓	✓	✓	✓	✓	✓	✓	✓	✓	✓	✓
	Bias (9)	?	-	-	?	-	-	-	?	-	-	✓	-	-	-	?	-	?	✓	✓	?	✓	-	-	-	-
	Study size (10)	✓	-	?	-	✓	?	✓	✓	?	-	✓	✓	✓	?	?	✓	✓	✓	?	✓	✓	✓	✓	✓	✓
	Quantitative variables (11)	✓	✓	✓	✓	✓	✓	✓	✓	✓	✓	✓	✓	✓	✓	✓	✓	✓	✓	✓	✓	✓	✓	✓	✓	✓
	Statistical methods (12.a)	-	✓	✓	✓	✓	✓	✓	✓	✓	✓	✓	✓	✓	✓	✓	✓	✓	✓	✓	✓	✓	✓	✓	✓	✓
	Statistical methods (12.b)	-	✓	✓	✓	✓	✓	✓	✓	✓	✓	✓	✓	✓	✓	✓	-	✓	✓	✓	✓	✓	-	-	✓	-
	Statistical methods (12.c)	-	-	-	-	✓	-	-	✓	-	-	✓	-	-	-	-	-	✓	-	?	-	✓	?	-	✓	-
	Statistical methods (12.d)	-	-	-	✓	✓	✓	✓	✓	-	✓	?	✓	✓	-	?	-	-	✓	-	✓	✓	✓	?	✓	?
	Statistical methods (12.e)	-	-	-	-	-	-	-	-	-	-	-	-	-	-	-	-	-	-	-	-	-	-	-	-	-
**Results**	Participants (13.a)	✓	-	✓	✓	✓	✓	✓	✓	✓	✓	✓	✓	✓	?	?	✓	✓	✓	✓	✓	✓	✓	✓	✓	✓
	Participants (13.b)	✓	-	-	?	?	✓	-	?	-	-	?	✓	-	-	-	✓	-	✓	✓	-	✓	✓	✓	✓	✓
	Participants (13.c)	✓	-	-	-	-	-	-	-	-	-	-	-	-	-	-	-	-	-	-	-	-	-	-	-	-
	Descriptive data (14.a)	?	✓	✓	✓	✓	-	?	✓	?	✓	✓	✓	?	?	?	-	✓	✓	✓	✓	-	?	✓	✓	✓
	Descriptive data (14.b)	✓	-	-	-	✓	-	-	-	-	-	?	-	-	-	-	-	✓	-	?	-	✓	-	?	✓	-
	Descriptive data (14.c)	n.a.	n.a.	n.a.	n.a.	-	n.a.	n.a.	n.a.	n.a.	n.a.	n.a.	n.a.	n.a.	n.a.	n.a.	n.a.	n.a.	n.a.	n.a.	n.a.	n.a.	?	n.a.	?	n.a.
	Outcome data (15)	✓	✓	✓	✓	✓	✓	✓	✓	✓	✓	✓	✓	✓	✓	✓	✓	✓	✓	✓	✓	✓	✓	✓	✓	✓
	Main results (16.a)	?	✓	✓	✓	✓	✓	✓	✓	✓	✓	✓	✓	✓	✓	✓	✓	✓	✓	✓	✓	✓	✓	✓	✓	✓
	Main results (16.b)	-	✓	✓	✓	✓	✓	✓	✓	✓	✓	✓	✓	✓	✓	✓	n.a.	✓	✓	✓	✓	✓	✓	✓	✓	✓
	Main results (16.c)	-	✓	n.a.	n.a.	✓	n.a.	✓	-	n.a.	?	✓	n.a.	n.a.	✓	?	n.a.	✓	n.a.	✓	?	-	✓	n.a.	n.a.	n.a.
	Other analyses (17)	-	✓	✓	✓	✓	-	✓	✓	-	✓	✓	✓	-	✓	✓	-	✓	✓	?	✓	-	-	-	✓	✓
**Discussion**	Key results (18)	✓	✓	✓	✓	✓	✓	✓	✓	✓	✓	✓	✓	✓	✓	✓	✓	✓	✓	✓	✓	✓	✓	✓	✓	✓
	Limitations (19)	✓	✓	✓	✓	✓	✓	✓	✓	✓	✓	✓	?	✓	?	✓	?	✓	✓	✓	✓	✓	✓	✓	✓	?
	Interpretation (20)	✓	✓	✓	✓	✓	✓	✓	✓	✓	✓	✓	✓	✓	✓	✓	?	✓	✓	✓	✓	✓	✓	✓	✓	✓
	Generalisability (21)	?	✓	-	✓	✓	✓	✓	?	✓	✓	✓	✓	?	✓	✓	-	✓	?	✓	-	-	✓	✓	?	-
**Other information**	Funding (22)	-	✓	?	✓	✓	-	-	-	?	✓	✓	-	-	-	-	-	-	✓	✓	-	✓	✓	-	✓	-
**Rate of criteria each study fulfilled (%)**		56.3%	62.5%	56.3%	71.0%	82.4%	68.8%	75.0%	71.9%	58.1%	71.9%	84.4%	74.2%	64.5%	50.0%	59.4%	53.3%	78.1%	80.6%	71.9%	68.8%	75.8%	69.7%	71.0%	78.8%	58.1%

✓: yes; -: no; ?: partially; n.a.: not applicable
